# Improving Sexual Assault and Sexual Harassment Prevention from the Bottom-up: a Pilot of Getting To Outcomes in the US Military

**DOI:** 10.1007/s11121-023-01577-3

**Published:** 2023-08-29

**Authors:** Matthew Chinman, Joie Acosta, Susan Bush-Mecenas, Sierra Smucker, Coreen Farris, Beverly Fortson, Pamela Imm, Andrea Lamont, Thomas Maguire, Laurie Martin, Abraham Wandersman, Amber Watson, Amanda Wicker, Andra Tharp

**Affiliations:** 1 RAND Corporation, 4570 Fifth Avenue, Pittsburgh, PA 15213 USA; 2 Department of Defense, Office of Force Resiliency, Washington, DC USA; 3 Wandersman Center, Columbia, SC USA

**Keywords:** Prevention, Sexual assault, Sexual harassment, Military

## Abstract

While the Department of Defense (DoD) has given increased attention and priority to preventing sexual assault and sexual harassment (SA/SH), it remains a problem. To build its prevention capacity, DoD piloted Getting To Outcomes^®^ (GTO^®^) from 2019 to 2022 at 10 military installations. GTO is an evidence-based planning and implementation support that has been used in many civilian contexts but has only recently been adapted for military SA/SH. The purpose of this study was to describe GTO use, identify its benefits and challenges, and discuss lessons the GTO effort yielded for prevention more broadly using a framework of organizational and program-level capacities needed for successful prevention in the military context, called the Prevention Evaluation Framework (PEF). GTO was piloted with 10 military installations (“sites”) representing all Military Services, plus the Coast Guard and National Guard. GTO is comprised of a written guide, training, and ongoing coaching. The pilot’s goal was for each site to use GTO to implement a SA/SH prevention program twice. Participants from each site were interviewed and data was collected on GTO steps completed, whether GTO spurred new evaluation activities and collaborations, and the degree of leadership support for GTO. Most sites completed all GTO steps at least once. Interviews showed that DoD participants believe GTO improved prevention understanding, planning, and evaluation capacity; strengthened confidence in chosen programs; and helped sites tailor programs to the military context. Barriers were the complexity of GTO, DoD personnel turnover, and the disruption that the COVID pandemic caused in sexual assault prevention program delivery. Many respondents were unsure if they would continue all of GTO after the coaching ended, but many believed they would continue at least some parts. According to the PEF, the GTO pilot revealed several additional prevention system gaps (e.g., need for leadership support) and changes needed to GTO (e.g., stronger leader and champion engagement), to support quality prevention. The military and other large organizations will need to focus on these issues to ensure prevention implementation and evaluation are conducted with quality.

## Introduction

While the Department of Defense (DoD) has given increased attention and priority to preventing sexual assault and sexual harassment (SA/SH), it remains a problem for the US military. DoD’s epidemiological estimates among active-duty Service Members (SMs) in 2021 show 8.4% of women (about 19,000) and 1.5% of men (about 17,000) experienced unwanted sexual contact in the past year. While a somewhat different metric for contact was used in 2021 which prevents comparison to 2018, numerically, these rates appear higher in 2021. Estimated rates of sexual harassment in 2021 among active-duty women increased from 2018, from 24 to 29%. The 2021 rate for men was similar to 2018, about 7%. Female victims are at increased risk of PTSD and other mental health disorders, attempted suicide, demotion in rank, and premature attrition from service (Rosellini et al., 2017). PTSD and mental health issues are nearly entirely explained by increased exposure to SA/SH (Jaycox et al., 2022). Reported less, male victims also face similar negative outcomes (Matthews et al., 2018; Millegan et al., 2016), and both male and female SA/SH victims are more likely to voluntarily leave the military than SMs who do not experience these crimes (Morral et al., 2021). DoD recognizes that comprehensive primary prevention is needed to stop SA/SH before it occurs.

### DoD Faces Multiple Challenges to Preventing SA/SH in the Military

First released in 2019 and updated in 2022, DoD developed the Prevention Plan of Action (PPoA). Drawing upon years of research on SA/SH prevention and implementation science, the PPoA outlines the requirements for a prevention system across several domains—e.g., infrastructure, leadership, and collaborations—for how each installation should conduct SA/SH prevention. To create a method by which to measure the elements of the PPoA in 2020, we developed the Prevention Evaluation Framework, an assessment tool describing what prevention should look like at military installations and military service academies (Acosta et al., 2022). The tool, based on literature and a panel of experts, describes organizational and program-level capacities needed to support military SA/SH prevention efforts, operationalized into 36 items in eight domains (see Table 1). Assessments using the PEF found that the implementation of effective prevention in DoD has been challenged by the organizational complexity of the Department of Defense and lack of prevention capacity at all levels (Acosta et al., 2021). For example, until very recently, DoD has had few designated positions whose sole function was to implement and evaluate SA/SH prevention activities. Furthermore, DoD’s infrastructure for prevention—e.g., leadership support, accountability, systematic evaluation, coordinated activities—has been underdeveloped and most prevention activities focus on building awareness rather than skills—e.g., brief lecture-based presentations (Office of Force Resiliency, 2022). Finally, another challenge is that SA/SH have many risk factors—e.g., perceptions of what peers believe is acceptable behavior, willingness to intervene on behalf of a potential victim, and alcohol and drug use (Tharp et al., 2013)—that can vary across the Department. Thus, multiple organizational capacities are needed both at the program (e.g., matching programming to documented need, continuously evaluating) and system (e.g., operate with accountability up and down the chain of command, provide leadership support) levels (Acosta et al., 2022) to address these challenges.

**Table 1:** Prevention evaluation framework—organizational factors

Factor	Definition
Leadership	Leaders use best evidence, monitor prevention activities, and hold subordinates accountable for their prevention work
Prevention workforce	Have sufficient numbers of training personnel who have regular contact with leadership
Collaborative relationships	Prevention personnel collaborate with each other inside the organization and with experts outside the organization
Data	Data is collected to document the specific nature of the problem locally and data is also used to track prevention impact
Resources	SA/SH prevention efforts have a dedicated budget for staffing, adaptation, implementation, evaluation, sustainability
Comprehensive approach to prevention	Prevention activities are evidence-based, target multiple risk factors across multiple ecological levels, and build skills as well as attitudes and knowledge
Quality implementation	Implementation processes are monitored for multiple elements of fidelity
Continuous evaluation	All prevention is regularly evaluated and improved

*SA/SH* sexual assault/sexual harassment

### Efforts to Build Capacity for Quality Prevention of SA/SH in the Military

DoD has taken multiple steps to build SA/SH prevention capacity, developing policies and guidance to support changes made at service headquarters (from the top-down), while simultaneously supporting capacity-building at individual installations (from the bottom-up).

#### Top-Down Efforts

In 2020, DoD issued the Policy on Integrated Primary Prevention of Self-Directed and Prohibited Abuse or Harm (DoDI 6400.09, 2020), which aims to establish a DoD-wide prevention system that makes data-informed decisions and implements research-based policies and interventions. In 2021, Secretary of Defense Austin directed multiple actions to build capacity of the prevention workforce, including establishing the Independent Review Commission on Sexual Assault in the Military (IRC), which conducted an independent, impartial assessment and made several recommendations about improving SA/SH prevention (Rosenthal, 2021). Specifically, the IRC recommended establishing a dedicated prevention workforce and equipping leaders with tools and competencies for prevention. In 2022, DoD started an effort to hire over 2000 new prevention personnel throughout the entire Department over the next 6 years. As the Department implements the Secretary of Defense approved IRC recommendations (Secretary of Defense memo, “Commencing DoD Actions and Implementation to Address Sexual Assault and Sexual Harassment in the Military,” September 22, 2021), it will require tools to support the development of comprehensive prevention efforts that are tailored to each military community.

#### Bottom-Up Efforts

The Department has also undertaken multiple efforts to build capacity from the ground up. This involved delivering webinars to the prevention workforce, developing a prevention workforce training, and developing and evaluating tools for the prevention workforce to use in prevention planning, implementation, and evaluation. The latter involved piloting Getting To Outcomes^®^ (GTO^®^) from 2019 to 2022 at 10 military installations. GTO is an evidence-based planning and implementation support process that has been used in many civilian contexts but has only recently been adapted for military SA/SH (Chinman et al., 2021; Ebener et al., 2022). The GTO pilot—this article’s focus—represents the first systematic effort by DoD to build SA/SH prevention capacity.

### Purpose and Contributions

The purpose of this study was to (1) describe how GTO was used at these installations; (2) identify benefits and challenges from using GTO and to what extent GTO was able to overcome those challenges and build prevention capacity; and (3) discuss lessons the GTO effort yielded for prevention more broadly. The contributions to prevention science are the lessons learned from employing a bottom-up capacity-building intervention in a military context, which represents a very large, and traditionally top-down organizational structure. To our knowledge, there has not been such an effort to build prevention capacity in the military using an implementation support like GTO before. To date, GTO has generally been evaluated in organizationally flat, community-based, and low-resource settings implementing youth prevention programming (e.g., Boys & Girls Clubs). In previous trials comparing organizations randomized to implement a prevention evidence-based program (EBP) on their own with youth or to implement the EBP with GTO, organizations using GTO implemented the EBP with higher fidelity (Chinman et al., 2016, 2018a, b) demonstrated better outcomes among participating youth (Chinman et al., 2018a, b), and were more likely to sustain the EBP after the end of the GTO support (Acosta et al., 2020). GTO sites made these gains despite facing organizational barriers such as a poor implementation climate (Cannon et al., 2019). In contrast, this study advances our understanding of the barriers and facilitators of using an implementation support like GTO in a large system, one of the first to do so.

## Methods

### Pilot Design, Participants, and Setting

As part of DoD’s effort to improve prevention capacity, we partnered with DoD’s Sexual Assault Prevention and Response Office (SAPRO) to apply GTO to 10 military installations (referred to as “sites”) representing all Services, Coast Guard, and the National Guard (sites are not named for confidentiality). Because this was a pilot, the focus was on understanding the facilitators and barriers and not on capturing impacts of individual participants in the programs run by each site. The sites—installations with hundreds to thousands of active-duty SMs and DoD civilian employees—varied widely and represent varied paygrades (from junior enlisted to senior leaders) and populations (age 18 to 50 +) of the military organization.

In 2018, SAPRO announced the availability of the opportunity for installations to participate and sites volunteered. Each site prioritized a different aspect of SA/SH prevention, planned different prevention activities, and convened a small GTO team (4–8 SMs and DoD civilian employees). Each installation was assigned two GTO “coaches” from a pool of 10 masters and doctoral-level prevention researchers trained in providing GTO coaching.

Sites participated in the pilot from 2019 to the middle of 2022 (two sites dropped out of the project after a few months). The pilot’s goal was for each site to complete two “GTO cycles.” Cycle 1 began with a needs assessment (GTO Step 1) and several planning, implementation, and evaluation activities. The interventions chosen were a mix of previously established evidence-based programs (e.g., Parent-Based Intervention, (Kuntsche & Kuntsche, 2016), those that were locally created but based on well-documented models in SA/SH prevention (e.g., bystander interventions, (Hoxmeier & Casey, 2022), and others that were locally created based on promising, but previously untested approaches. See Table 2 for a list of the interventions by site. After the intervention was delivered to one cohort of SMs, the GTO coaches helped analyze the data and led the GTO site team through a quality improvement process (Step 9) to revise the intervention for the next cohort of SMs (Cycle 2). Sites were then asked to consider how to sustain the intervention (Step 10). Table 3 shows how each site performed the various practices in each GTO step, to implement their chosen SA/SH program.

**Table 2:** GTO progress, impact, and support from leadership across DoD sites

Site	Program	GTO steps completed		Leadership support 1 = Low 2 = Medium 3 = High	GTO’s impact on programming: • GTO led to the program’s creation • GTO was used to revise the program • Program pre-dated GTO	GTO’s impact on process/implementation evaluation: • GTO led to the evaluation’s creation • GTO was used to revise the evaluation • Evaluation pre-dated GTO	GTO’s impact on outcome evaluation: • GTO led to the evaluation’s creation • GTO was used to revise the evaluation • Evaluation pre-dated GTO	GTO’s impact on partnerships: • GTO created new collaborations • GTO revised existing collaborations • Collaborations pre-dated GTO
		GTO cycle 1	GTO cycle 2					
1	Maintaining Respect in the Workplace	Steps 1–10	Steps 1–10	3	Revised	Revised	Created	Created
2	Take A Stand	Steps 1–6	None	3	Pre-dated	Revised	Pre-dated	Revised
3	Sexual harassment; alcohol misuse; company climate	Steps 1–10	Steps 1–10	3	Created	Created	Created	Created
4	Building a Better Workplace	Steps 1–10	None	2	Revised	Created	Created	NA
5	Parent-based Intervention	Steps 1–10	Steps 1–10	2	Created	Created	Created	Created
6	Sex Signals; Got Your Back	Steps 1–10	None	3	Pre-dated	Created	Created	NA
7	TimeOut	Steps 1–5	None	*	Created	NA	NA	Created
8	Bystander intervention training	Steps 1–10	None	3	Created	Created	Created	Revised
9	Maintaining Respect in the Workplace	Steps 1–6	None	1	Revised	Created	Created	Created
10	NA	None	None	1	NA	NA	NA	NA

*NA* no activity. *Missing

**Table 3:** GTO manual information and practices performed by GTO teams

**GTO step**	**Step-specific information**	**Example practices by step**
1. Needs: *What are the needs to address and the resources that can be used?*	Information about how to conduct a needs and resources assessment using surveys and administrative data sources the DoD already collects	Sites staff reviewed data sources such as Workplace and Gender Relations Survey of Active Duty Members, the Defense Organizational Climate Survey, and incidents of SA/SH to learn about the needs of their Service Members
2. Goals and outcomes: *What are the goals & desired outcomes?*	Tools for creating measurable goals & desired outcomes	Each site developed their own broad goals and “desired outcomes”—statements that specify the amount and timing of change expected on specific measures of knowledge, attitudes, behavior regarding SA/SH
3. Best practices: *Which evidence-based programs can be useful in reaching the goals?*	Overview of the different types of evidence-based SA/SH programs and where to access information about them (e.g., Clearinghouse for Military Family Readiness)	The GTO team, in concert with installation leaders at the site, chose a specific SA/SH prevention program to implement
4. Fit: *What actions need to be taken so the selected program fits the community context?*	Tools to help sites ensure chosen SA/SH programs are consistent with military culture	Each site reviewed possible SA/SH prevention programs for how they would fit within their installation and made adaptations to improve fit
5. Capacity: *What capacity is needed for the program?*	Assessment tools to help sites ensure there is sufficient organizational, human and fiscal capacity to conduct the SA/SH program (e.g., senior commander support)	Each site assessed their own capacity to carry out their chosen SA/SH prevention program and made plans to increase capacity when needed
6. Plan: *What is the plan for this program?*	Information and tools to plan the SA/SH program activities in detail	Each site conducted concrete planning for implementing their chosen SA/SH prevention program (e.g., who, what, where, when)
7. Process evaluation: *How will the program implementation be assessed?*	Information and tools to help sites plan and implement a process evaluation of their SA/SH program	Each site collected data on various aspects of implementation (e.g., fidelity, attendance, satisfaction) to assess program delivery and reviewed that data after implementation
8. Outcome evaluation: *How well did the program work?*	Information and tools to help sites implement an outcome evaluation, including lists of measures commonly used to assess SA/SH prevention	Each site collected pre-post participant outcome data on actual behavior (e.g., number of times intervened as a bystander) as well as on mediators such as attitudes and intentions (e.g., belief about intervening, intentions to intervene)
9. Continuous quality improvement: *How will continuous quality improvement strategies be used to improve the program?*	Tools to prompt sites to reassess GTO steps 1–8 to stimulate improvement plans for their SA/SH program	Each cycle, each site reviewed evaluation data, previous planning decisions, and tools completed before implementation and made concrete changes for the next implementation
10. Sustainability: *If the program is successful, how will it be sustained?*	Ideas to use when attempting to sustain an effective program (e.g., ensuring program becomes part of the installation’s regular routine)	Each site considered various sustainability factors such as securing adequate funding, staffing, and installation command buy-in, to make it more likely that their SA/SH program would be sustained

### Getting To Outcomes for Sexual Assault and Sexual Harassment Prevention in the Military

GTO is an implementation support process of 10 steps any organization should progress through (six for determining needs and planning; three for evaluation and improvement; one for sustainment) and then builds capacity with written tools, training, and ongoing coaching to help those organizations complete those steps with quality, as applied to their intervention. As defined by the Expert Recommendations for Implementing Change (ERIC, (Powell et al., 2015), GTO combines strategies of training and educating stakeholders, providing facilitation, multiple evaluative and iterative strategies, adapting and tailoring to the context, and supporting clinicians/practitioners (Chinman et al., 2013; Matthew Chinman et al., 2008a, b). GTO’s capacity-building is rooted in social cognitive theories of behavioral change (Ajzen & Fishbein, 1977; Bandura, 2004; Fishbein & Ajzen, 1974) in which practitioners are asked to be active learners—i.e., GTO establishes expectations and gives opportunities and guidance for practitioners to carry out for themselves the best practices that GTO specifies across the 10 steps. GTO employs multiple approaches advocated for by (Beidas et al., 2022) to accommodate site characteristics, namely empowering sites to choose interventions that fit their needs (GTO Step 3, Table 2), offering a menu of support options (assisting with data analysis, briefing senior leaders), and using facilitation for GTO’s coaching model in order to be adaptive to site needs. GTO has been applied to several content domains including the prevention of teen pregnancy, underage drinking, youth drug use, Veteran homelessness (Chinman et al., 2004; Ebener et al., 2017; Hannah G. et al., 2011; Imm et al., 2007; Mattox et al., 2013), and was tailored to the military context in multiple ways. We adapted the GTO *manual* (and the online, streamlined version)—i.e., defining SA/SH drivers, describing specific evidence-based SA/SH prevention programs, and presenting evaluation measures relevant for SA/SH prevention (e.g., intentions to intervene in a risky situation, (Chinman et al., 2021; Ebener et al., 2022). The manual was based on a literature review and input from experts in prevention of SA/SH and military SA/SH. The manual also presented data on SA/SH prevalence from DoD’s bi-annual Workplace Gender Relations Assessment (see Table 3 for a list of GTO information applied to SA/SH in the military, by step). Each site’s GTO team received a full day *training* to introduce them to the 10 steps, familiarize them with the GTO manual, and practice using GTO tools by working through a fictional example of a military installation trying to improve their prevention. After the training, there were bi-weekly *coaching* meetings at each site to complete the steps.

### Data Collection

#### GTO Coach Self-Reported Progress Form

GTO coaches from each site were asked to complete a short, structured form asking for site name, prevention program name (and whether the program’s start was facilitated by GTO or pre-dated GTO), GTO steps completed, whether the site used GTO to conduct new evaluation activities, and whether GTO spurred any new collaborations. In previous studies, GTO has demonstrated success in helping practitioners start new program evaluations and forge new partnerships (Chinman et al., 2013; Matthew Chinman et al., 2008a, b) and these are key domains in the Prevention Evaluation Framework described above. This self-report was checked by two researchers by comparing the self-reports with online document registries containing completed GTO tools and training materials for each site.

#### Site Interviews

We utilized a multiple case study approach (Merriam, 1998; Yin, 2009), purposively sampling and recruiting respondents most closely involved in GTO at each site—i.e., GTO Site Team members. Using a common interview guide, GTO team members were interviewed about the following: what did and did not work in utilizing GTO to plan, implement, and evaluate prevention activities; the likelihood of sustainability and compatibility of GTO with the military; leadership support, communication, and collaboration across functional areas or helping agencies; personnel support, capacity, and turnover; the role of the site champion (i.e., SM or DoD civilian employee committed to shepherding the GTO prevention processes from start to finish); site culture; and recommendations for supporting sustainability of GTO. The interview protocol was developed by the authors, who have extensive expertise with GTO, in collaboration with SAPRO. The team also included DoD GTO coaches who reviewed questions for relevancy.

Interviews were conducted by three researchers who were not involved in coaching. We conducted 21 semi-structured discussions with leaders and team members at nine sites. One site was not available. Two sites ended their GTO participation early and were interviewed soon after they ceased participation (mid 2020), and the remaining eight sites were interviewed in mid to late 2021 in eight group discussions with key site participants (*n* = 14 individuals across all group discussions), documented through detailed notetaking. Six months later, we revisited the eight sites, conducting an additional 13 individual discussions, which were audio recorded and transcribed. We removed all identifying information in documentation and quotations presented in this report to preserve the anonymity of the participants.

### Data Analysis

#### GTO Coaches Self-Reported Progress Form

Forms were synthesized into Table 2 to systematically describe each site’s progress in utilizing GTO—e.g., number of GTO cycles and steps completed. A GTO step is “completed” when the tools from the GTO manual are finished and of sufficient quality as deemed by the GTO coach. Cycle 1 was considered complete when a site proceeded through GTO Steps 1–6, conducted the intervention with a cohort of SMs, and then used evaluation data to complete Steps 7–9. Cycle 2 was considered complete when the site conducted the intervention, collected data, and engaged in quality improvement a second time, followed by GTO’s sustainment step (Step 10). Each coach’s responses about GTO’s impact on the program, the process and outcome evaluation of that program, and new partnerships were coded by the lead author and double coded by the second author, both experts in GTO. The coding scheme for all four items was as follows: GTO led to the *creation* of the program/evaluation/partnership; GTO was used to *revise* existing program/evaluation/partnership; or the program/evaluation/partnership *pre-dated* the use of GTO. Also, for certain items at certain sites, there was *no activity*, meaning that the site did not progress through the relevant GTO Steps related to program, evaluations, or partnerships.

#### Site Interviews

All transcripts and notes were coded in Dedoose qualitative data analysis software. We identified a deductive coding scheme including descriptive (e.g., Services), thematic (e.g., leadership support), and analytic (e.g., level of sustainability) codes (Saldana, 2021). To assess inter-rater reliability across coders, two researchers coded 10% of interviews and compared the level of agreement in coding at 75% agreement or above across the different codes (O’Connor & Joffe, 2020). Any disagreements in coding were discussed and adjudicated to ensure intercoder reliability. These two researchers coded the remainder of the transcripts. Finally, we conducted a thematic analysis using analytic memoing to identify common patterns in the benefits and challenges of GTO and likelihood of continued use of GTO (Braun & Clarke, 2006). We then summarized coded data at the site level utilizing a cross-case (i.e., site) meta-matrix to examine patterns between the benefits, challenges, and sustainability of GTO with various aspects of organizational structure and dynamics (Bush-Mecenas & Marsh, 2018; Miles et al., 2018). To strengthen the validity of our findings, we triangulated data across interview transcripts and notes as well as GTO documentation, where possible (Denzin, 1978; Patton, 1999). In our analysis process, we attempted to craft rival hypotheses (alternative theories, research bias, threats to validity) and real-world rival hypotheses (alternative theories, implementation issues), to test and validate our analyses (Yin, 2013).

Given the importance of leadership support, we specifically coded this domain, by site, as low (= 1), medium (= 2), or high (= 3) (see Table 2). High included senior leadership being aware of GTO and projects from the beginning—e.g., asking what step the GTO Team was on or tracking project outcomes. Medium was some general awareness, but less supportive—e.g., a leader expressing support for the prevention program, but being unaware of the connection to GTO. Low was where respondents experienced limited to no buy-in or support from senior leaders. The leadership support data was also added to Table 2.

## Results

### GTO Progress and Leadership Support

As shown in Table 2, GTO coaches reported that 9 sites (90%) completed GTO steps 1–6, six sites (60%) completed all 10 steps of GTO (completing a full cycle of GTO) and three sites (30%) completed all ten steps twice—i.e., completing two full cycles of GTO. Seven sites (70%) used GTO to either implement a new prevention program or revise a previously chosen program. Eight sites (80%) used GTO to either begin or revise process and outcome evaluations of these programs. Leadership support varied, with five sites rated at the highest level, one site rated at the mid-level, and two sites at the lowest level. No site rated at the lowest level conducted a second cycle of GTO.

### Qualitative Data

#### Benefit Sites Accrued from Using GTO

##### Improved Prevention Understanding

Respondents at six sites noted that GTO improved their understanding of prevention and evidence-based programs and strengthened their capacity to implement prevention activities. One respondent shared,

I think [the GTO] process has really expanded my knowledge … The process itself and being able to go through the tools to complete with our advisors, being able to ask questions about the process, getting advice ... It has really helped us to understand primary prevention and because of that we were able to develop a prevention strategy.

Also, respondents at half of the sites noted that because of using GTO, they had moved from a focus on compliance (e.g., enforcing participation in annual online training) toward a focus on understanding whether the prevention efforts being used were effective.

(Before GTO) there was nothing that really got off the ground in terms of prevention. With the GTO project, we actually could go step by step in determining whether or not something was actually effective.

##### Improved Planning

Respondents at seven sites reported improving their knowledge specifically about program design and planning. Prior to GTO, respondents indicated there was no strategic prevention planning process in place, and that prevention was planned in a reactive or haphazard manner. One site described this process as their “throw spaghetti on the wall and see if it sticks approach.” GTO coaches provided a structure that respondents described as building their understanding of what constitutes quality prevention. Others commented on how GTO helped them to “expand the seats around (their) prevention table” and attend more readily to coordination between the personnel responsible for prevention and others at the installation that could provide useful insights, data, referrals, and other resources.

##### Improved Evaluation Capacity

Four sites noted that, prior to using GTO, they had engaged in very limited or no evaluation of prevention activities. These sites reported that gathering data about program effectiveness helped them adapt and improve their programs. For example, at one site, the GTO team was able to document the relationship between program dosage and prevention knowledge among program attendees (e.g., what constitutes sexual harassment), which strengthened leader support. Respondents at five sites reported that they were able to draw upon data and resources gathered through GTO to lessen the burden of compliance activities. For example, administering feedback surveys provided useful data and information to include in program reports or process evaluations.

##### Strengthened Confidence in Chosen Programs

At most sites, respondents noted that using the GTO process had helped increase confidence in the selected programs, and prevention activities in general, among the implementation team members as well as leadership. For example, respondents described the importance of being able to prove their prevention efforts are effective because these efforts compete with other duties. Sites indicated that GTO specifically helped by supporting the selection of evidence-based programs and implementation of process and outcome evaluations. These efforts were described by one site as adding “richness and validity to the services (the installation) provides” and helping to motivate personnel by helping them to feel proud of their work quality.

##### Assistance Tailoring Programs to the Military Context

A third of sites noted that using the GTO process helped site participants to adapt and modify programs for the military context. One respondent stated that “…[GTO] allowed my team to make an established program more efficient and effective to …[our enlisted personnel].” Another site described how the GTO process helped them to better tailor their program to younger soldiers, who they found challenging to reach using prior interventions.

#### Champions Played an Important Role

The respondents stated that champions—SMs and DoD civilians in mid-level leadership roles—played a crucial role in maintaining a commitment to GTO and, in some sites, ensuring new site participants gained GTO training. At the site level, champions often were responsible for communicating the benefits of GTO and the efficacy of prevention programs up the chain of command to senior leaders. Champions also often served as consistent team members and advocates for the work, especially at sites where there was high personnel turnover on the GTO teams. In some sites, champions also took responsibility for ensuring that new personnel were trained in or guided through the GTO process. This was especially important where responsibilities for prevention programs were assigned to individuals not familiar with program planning and/or prevention practice. Like GTO coaches, champions also played an important role in maintaining enthusiasm, commitment, and accountability among team members.

#### Challenges to Using GTO

##### Complexity of GTO

At about half of the sites, respondents shared that GTO was complex and more academic than their typical procedures. It took time and effort to build the capacity (i.e., experience, knowledge, and skills) of site personnel to use GTO and to onboard new personnel following turnover. While respondents were enthusiastic about their own growth and learning through GTO training and use, they noted that the process might be less accessible to individuals with less experience.

##### Turnover and Time

Turnover occurred frequently at every site and was consistently described as a major challenge to implementing and sustaining GTO. Several sites noted that even after GTO helped build site prevention capacity, turnover necessitated starting over with new team members, which slowed the GTO work. At every site, respondents also noted that it was often a challenge to fully and consistently engage due to competing priorities and demands. This was especially true where personnel were assigned to participate in prevention activities as a collateral duty—which was the case at almost every site.

##### COVID

Challenges in GTO implementation were further exacerbated by the COVID-19 pandemic. Respondents across all sites described how the pandemic slowed their GTO and prevention work and, in some sites, hurt implementation. For example, three sites had to change to online program delivery or reduce the number of units receiving the prevention program. The elongated timeline for the GTO work caused by the pandemic also amplified the challenges around personnel turnover. One respondent described, “the biggest challenge we had is because of the COVID. Everything went to a standstill….we lost some members [of our GTO team]… and some of the members got new job duties…” While scheduled turnover (i.e., movement to other installations) is characteristic of military contexts, the slowing of the GTO process during the pandemic meant that teams experienced greater turnover than expected.

#### Likelihood of Sustaining GTO to Support Quality Prevention

Many respondents were unsure whether their site would continue utilizing GTO after the coaching ended. Overall, sites where respondents reported greater benefits of GTO were more likely to report a higher likelihood of sustaining GTO. However, benefiting from GTO was not a guarantee for sustaining GTO. For example, personnel capacity and shortages could derail sustainability, as one respondent articulated:

I would say [we may not continue to use GTO]. They might use aspects of the project, of the model. They will probably, most definitely use the program that was crafted out of this model, but I don’t know if they would access this model to look at something new and different, because there’s nobody there onsite [due to personnel turnover].

In a few sites, respondents noted that they would keep using GTO, or elements of GTO, in their individual work planning processes. About half also noted that their sites might continue to use specific activities learned through GTO, such as fielding surveys and feedback forms to capture evidence of program effectiveness.

## Discussion

This project was the first systematic effort to build capacity for SA/SH prevention in the US military using a bottom-up approach like GTO. Almost all participating sites were able to implement a prevention program, some with multiple cohorts of SMs, despite COVID-19 restrictions. Although mostly qualitative, the data is consistent with previous GTO randomized and quasi-experimental trials in civilian contexts in which those who were more engaged in the GTO process experienced greater improvements in their programming and had larger gains in capacity (Acosta et al., 2013; Chinman et al., 2013; Matthew Chinman et al., 2008a, b).

The challenges that GTO faced in the 10 sites reveal larger prevention systems issues within DoD that have implications for prevention across the Department. These larger issues are not just relevant for the US military. They could as easily apply to the public school systems, who have also been looked to for implementing various prevention programs, but often have not been able to do so with any impact (Chinman et al., 2019). The eight domains of the Prevention Evaluation Framework (in *italics* below) are a useful guide for how to view these key systems issues of prevention—which of these a bottom-up approach like GTO can impact—and how the lessons learned from the GTO pilot can inform plans for the future of prevention in DoD and in large organizations in the civilian sector as well as what changes are needed for implementation strategies like GTO to make those strategies more accommodating to sites.

While the top DoD *leadership* (e.g., Secretary of Defense Austin) strongly endorses a robust prevention system,^1^ leaders lower in the chain of command (i.e., base commanders) often have a more direct impact. In the pilot, sites with more engaged, knowledgeable installation leaders used GTO more comprehensively, operated with greater accountability, and were more likely to endorse continuing to use GTO. Although many correctly point to “leadership” as being an important predictor of evidence-based practice uptake (Hannes et al., 2010; Vroom et al., 2021), this study highlights the need for all organizations, including DoD, to activate the “middle” leadership layer. Included in this layer are champions, those who support, market, and support program implementation and help to overcome resistance to prevention efforts in an organization (Bonawitz et al., 2020). This study showed how champions were effective—e.g., by communicating the benefits of GTO and prevention up the chain of command and providing consistency in the face of GTO Team member turnover. As is in this study, organizations that have both champions and supportive leadership appear better poised to conduct effective prevention.

Given the importance of leadership and champions, additions to the GTO implementation strategy could include securing preliminary agreements up front and making changes to the construction of the GTO implementation teams. In the current and past projects (Chinman, Acosta, et al., 2018; Chinman, Ebener, et al., 2018), the GTO implementation teams have been made of naturally emerging champions and individuals who were directly responsible for implementation who would then reach out to leadership for assistance. In these projects, the participating organizations were much flatter than DoD. Thus, in the current project, while certain champions did emerge and facilitate, it would likely improve implementation if GTO coaches engaged in a more intentional process of identifying champions a priori. Including key opinion leaders across multiple levels as part of the team, and strategically identifying them through a diffusion of innovation lens (i.e., early adopters) and matching their characteristics to contextual factors of the organization as recommended by (Bunce et al., 2020), could improve leadership support. Furthermore, studies have shown that *multiple* champions are often needed for successful implementation (Damschroder et al., 2009; Shaw et al., 2012; Soo et al., 2009), especially in a hierarchical organization like DoD with multiple levels of command. This approach could help ameliorate the fact that the senior leaders who volunteered the participation of their sites were not involved in GTO or the implementation of the chosen intervention at the site in their command—a circumstance that is common especially in large organizations.

Another critical factor for GTO in these sites and across DoD is the availability of a dedicated, trained *prevention workforce*. While GTO was able to successfully train GTO teams at each site, turnover, busy schedules, and the lack of dedicated personnel were constraints over time. Whether asking middle school teachers to incorporate drug prevention into their health class or asking DoD sexual assault *response* personnel to add prevention to their portfolio, organizations attempting prevention will be less successful without qualified and dedicated prevention personnel—with or without implementation support approaches such as GTO. The time required and complexity of GTO were drawbacks mentioned by sites; however, a qualified and dedicated workforce may be more effective in utilizing such support. The effort underway by DoD to hire new dedicated prevention personnel— ~ 350 as of May 2023, ~ 2000 planned (Department of Defense, 2022)—is an opportunity, but must be done with care as new staff who are tasked to implement a new kind of service can be siloed or unwelcome. For example, the Department of Veterans Affairs (VA) hired and deployed 1200 “Peer Specialists,” individuals with mental illnesses and substance abuse disorders who are trained to use their experience to help other Veterans with similar problems (M. Chinman et al., 2008b). Using implementation science methods, many researchers have documented how traditional VA staff have been extremely hesitant about incorporating this new kind of provider, despite evidence showing they improve outcomes and are greatly valued by Veteran patients (e.g., Chinman et al., 2006). Implementation strategies, such as collaboratively planning the new service, have been helpful in mitigating these challenges (Chinman et al., 2010) and would likely be useful in deploying the prevention workforce in DoD.

After the foundational domains of leadership and the prevention workforce, three additional domains—*collaborative relationships, data,* and *resources*—must be considered. GTO was able to make some impact on all three: brokering collaborations across silos, ensuring program evaluation data was collected and analyzed to support data-driven decisions, and helping GTO site team members to request more resources. However, to truly have a functional prevention system, organizations like DoD must integrate previously siloed efforts—including having personnel tackling different related domains (e.g., sexual assault, alcohol), coordinating their programming, and sharing data. While adequate resources are needed to support personnel and programming (e.g., Chinman et al., 2012), this study shows that resources do not exist in a vacuum, but are tied to engaged and supportive leadership, which in turn often requires ongoing access to data showing the impact of prevention on outcomes.

Lastly, the final three domains—*comprehensive approach to prevention, quality implementation,* and *continuous evaluation*—all relate to the conduct of prevention activities on the ground. GTO’s training, tools, and coaching were able to support better quality prevention than had previously been implemented. GTO guidance strongly encourages organizations to implement comprehensive prevention that is consistent with evidence; however, in the military, that was difficult. Most evidence-based prevention programs were developed outside the military and must be adapted (Acosta et al., 2021; Perkins et al., 2016), requiring a higher level of skill among those doing the adapting. Implementation supports like GTO can help, as shown in this study, but having a larger number of military-tested, evidence-based programs available would greatly enhance adoption. Implementing with quality and conducting continuous evaluation—key elements of any prevention effort—often requires a culture that genuinely uses the results of these activities (i.e., evaluation data) and rewards them. As demonstrated, GTO was able to support these activities, but ultimately, meeting the demands of these three domains across the entire military will largely be dependent on the other domains of Prevention Evaluation Framework—e.g., supportive leadership, appropriate workforce, and resources.

### Limitations and Future Research

Although the first to evaluate implementation support—GTO—for prevention in the military, this study used a small number of sites and did not assess SM outcomes but focused on the impacts of GTO on sites’ prevention capacity and performance. Future studies in the military, and other large organizations, should include large, cluster-randomized trials where sites tasked with prevention are randomized to receive GTO or not. Similar to GTO studies in community settings (Acosta et al., 2013; Chinman, Acosta, et al., 2018; Chinman, Ebener, et al., 2018), such trials should assess site- and implementer-level characteristics, implementation outcomes (e.g., fidelity, dose), outcomes of individual participants, while adding social network analyses to assess impacts of champions.

## Conclusion

We piloted GTO at 10 military bases across DoD to support better SA/SH prevention. While there were certain challenges (time, complexity, COVID), GTO was generally successful at improving the quality of specific prevention activities. However, the use of GTO revealed that successful implementation of prevention in a military context (and likely any organizational context) also requires a prevention infrastructure highlighted by the first five elements of the PEF—e.g., leadership, prevention workforce, collaboration, data, and resources. Given these elements were nascent during the GTO pilot, the military (or any organization) will need to focus on these issues to ensure prevention implementation and evaluation are conducted with quality—the final three elements of the PEF. Also, while GTO has features that accommodate setting characteristics recommended by (Beidas et al., 2022), this study revealed changes needed within GTO to better accommodate large organizations like the military, including more intentional engagement of leadership and identification of champions.

## Data Availability

The data from this study is available upon request from the authors.

## Footnotes

1 https://youtu.be/ITQhikaQkek
